# Effect of Keloid Properties on Treatment Efficacy: A Systematic Review

**DOI:** 10.1097/DSS.0000000000004256

**Published:** 2024-06-14

**Authors:** Vazula Bekkers, Paul Barsoum, Qi Yin, Frank Niessen, Paul van Zuijlen, Oren Lapid, Martijn van Doorn, Albert Wolkerstorfer

**Affiliations:** * Department of Dermatology, Erasmus Medical Center, Rotterdam, The Netherlands;; † Department of Dermatology, Amsterdam University Medical Center (AUMC), Location University of Amsterdam, Amsterdam, The Netherlands;; ‡ Department of Plastic, Reconstructive and Hand Surgery, AUMC Location Vrije Universiteit Amsterdam, Amsterdam, The Netherlands;; § Burn Center and Department of Plastic, Reconstructive and Hand Surgery, Red Cross Hospital, Beverwijk, The Netherlands;; ‖ Pediatric Surgical Center, Emma Children's Hospital, AUMC Location University of Amsterdam, Amsterdam, The Netherlands;; ¶ Amsterdam Movement Sciences Institute, AUMC, Amsterdam, The Netherlands

## Abstract

**BACKGROUND:**

The efficacy of keloid treatment in randomized studies is highly variable. However, no systematic review has been performed to evaluate the effect of different keloid properties on treatment efficacy.

**OBJECTIVE:**

To identify clinically relevant keloid properties that may influence treatment efficacy.

**MATERIALS AND METHODS:**

An electronic database search was conducted. Two reviewers independently selected randomized controlled trials (RCTs) and performed a methodologic quality assessment using the Cochrane risk-of-bias 2.0 tool.

**RESULTS:**

One thousand five hundred twenty studies were screened, and 16 RCTs, involving 1,113 patients, were included. The authors found lower efficacy in older keloids (*n* = 3), keloids located on the chest, extremities, pinna, and shoulder (*n* = 3), larger keloids (*n* = 2), lower baseline Vancouver Scar Scale score (*n* = 1), and keloids with history of recurrence (*n* = 1). Overall, most studies had a high risk of bias.

**CONCLUSION:**

Only a minority of studies specifically addressed keloid properties, which makes comparisons between studies challenging. The authors' results suggest that keloid location, duration prior to treatment, size, history of recurrence, and severity are clinically relevant keloid properties that affect treatment efficacy. Further studies are crucial to corroborate the authors' findings, establish a clinically relevant keloid classification, and ultimately develop an evidence-based treatment algorithm that takes these properties into account.

## Abstract 2

Supplemental Digital Content is Available in the Text.

Keloid, derived from “cheloides,” the Greek word for “crab's claw,” is a fibroproliferative scar that expands beyond the initial border of injury and rarely shows spontaneous regression. These pathologic scars can cause severe pain, pruritus, and functional or aesthetic complaints, which can decrease patients' quality of life.^1^

The reported clinical efficacy of keloid treatments is highly variable and may strongly depend on keloid and patient characteristics. However, reaching consensus on a standardized keloid classification system based on the most relevant clinical properties remains challenging. Ideally, this classification should be based on high level evidence that shows the impact of specific properties on treatment efficacy. This could be a crucial step towards developing evidence-based guidelines for selecting the most efficacious treatment for individual keloid patients.

However, to date, no systematic review has been performed to evaluate the evidence regarding the impact of different keloid properties on treatment efficacy. This systematic review aimed to assess the impact of the various keloid properties on treatment efficacy.

## Methods

A comprehensive electronic literature search was performed by a Biomedical Informatics Specialist in Cochrane Central Register of Controlled Trial, Embase, Google Scholar, Medline ALL, and Web of Science Core Collection (See Supplemental Digital Contents 1 and 2, http://links.lww.com/DSS/B456 and http://links.lww.com/DSS/B457). This systematic review was registered in PROSPERO (CRD42023451685), and the PRISMA 2020 checklist was followed (see Supplement Digital Contents 1 and 2, http://links.lww.com/DSS/B456 and http://links.lww.com/DSS/B457).

Duplicates were removed, and titles and abstracts were screened for eligibility independently by 2 reviewers (V.B.; P.B). Hereafter, full-text articles were assessed for eligibility. Randomized controlled trials (RCTs) were included if the full-text was published in English from inception to August 2023, and if they assessed the efficacy of any keloid treatment in patients of all ages, with at least 1 keloid property analyzed. Studies were excluded if they did not provide separate analyses for keloids when hypertrophic scars were also included.

Standardized data extraction and methodologic quality assessment of the included studies were performed independently by V.B. and P.B. Discrepancies between reviewers were discussed and resolved by consensus and if necessary, discussed with A.W. The collected data included the analyzed keloid properties, treatment efficacy, primary outcome measure, total no. of keloids and patients, and keloid therapies used. Methodologic quality was assessed using the Cochrane risk-of-bias 2.0 tool (ROB 2.0), and figures of the methodologic quality assessment were created with Robvis.^2^

## Results

**Figure 1.**
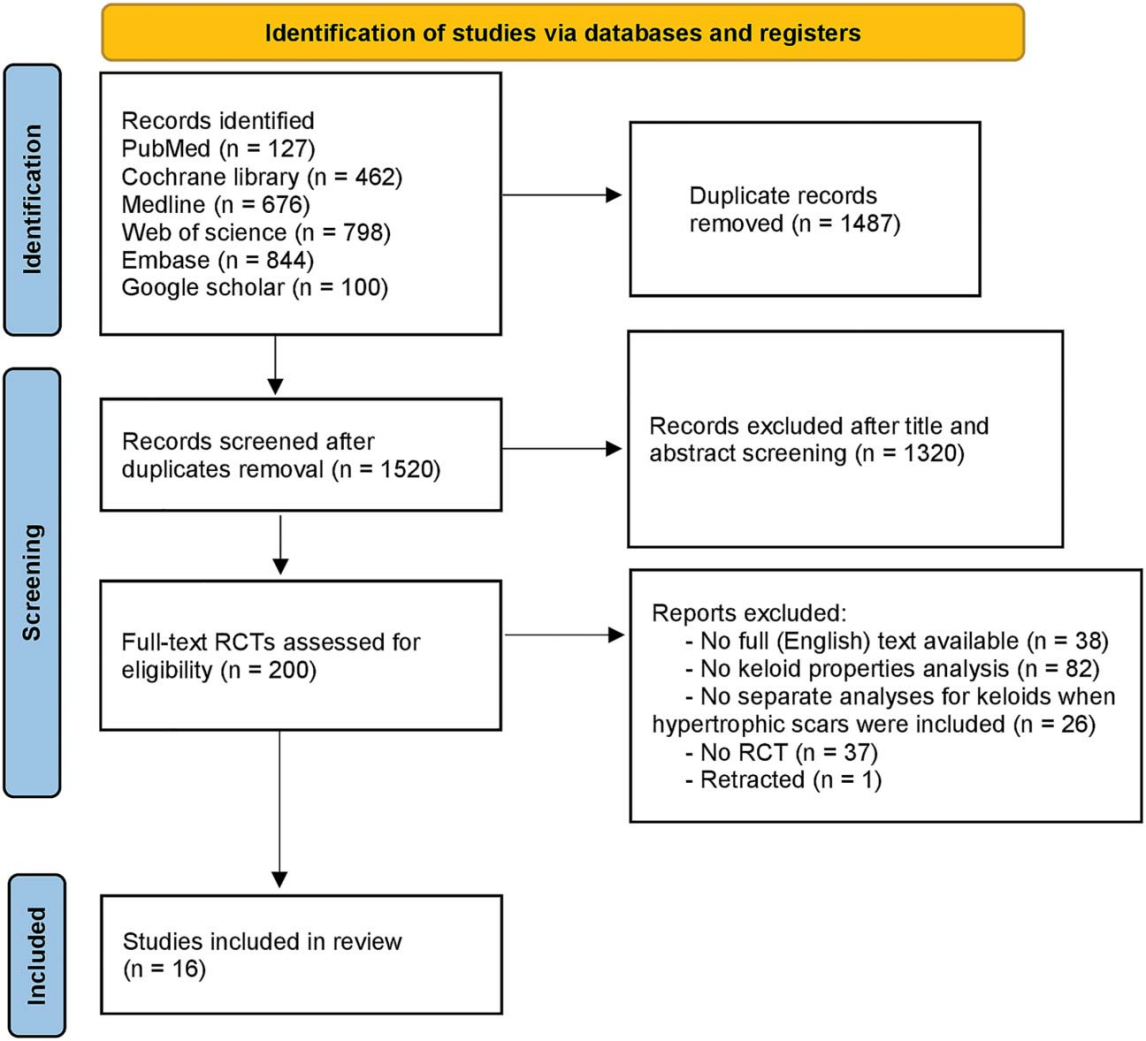
Study flow diagram resulting in 16 included studies.

The authors' literature search identified 1,520 studies, of which 16 studies with a total of 1,113 patients were included for data assessment (Figure 1 and Table 1).

**TABLE 1.** Results of the Included Studies

Study	Keloid Properties	Treatment Groups	Results	Outcome Measure	Relevant Study Characteristics (*n* = No. Keloids)
Abdel-Meguid et al., 2015	Duration, size	A: IL cryosurgery; B: contact cryosurgery	Duration: NS.	Keloid height	Duration: ≤2 years (*n* = 48), > 2 years (*n* = 18)
			Size: smaller keloids had a better response to contact cryosurgery, while size of the keloids did not significantly affect the response to intralesional cryosurgery		Size: small <1 cm^2^ (*n* = 41), medium 1–5 cm^2^ (*n* = 21), large >5 cm^2^ (*n* = 4)
					Total no. keloid patients: 33
Albalat et al., 2022	Duration, skin type	A: IL TCA; B: IL verapamil; C: IL 5-FU; D: IL PRP	Fitzpatrick skin type and duration: NS.	POSAS score	Mean duration (months): 4 (*n* = 40), 5 (*n* = 40), 4 (*n* = 40), 5 (*n* = 40)
					Fitzpatrick skin type: type 3 (*n* = 109), type 4 (*n* = 45), type 5 (*n* = 6)
					Total no. keloid patients: 160
Aluko-Olokun et al., 2014	Location	A: IL TCA; B: excision + radiotherapy	Location: higher cure rate on cheek, forehead, submandibular, and lip with TCA compared to the pinna. NS between different locations in excision + radiotherapy group	Keloid height and recurrence	Location (TCA group): pinna (*n* = 13), cheek (*n* = 16), forehead (*n* = 7), submandibular (*n* = 9), lip (*n* = 11)
					Location (excision + radiotherapy group): pinna (*n* = 12), cheek (*n* = 13), forehead (*n* = 7), submandibular (*n* = 12), lip (*n* = 9)
					Total no. keloid patients: 107
Belie et al., 2021	Location	A: IL verapamil; B: IL TCA monotherapy	Location: significant decrease in pain and pruritus in keloids located on the head, neck, and trunk with TCA, at resp. the 2^nd^ and 3^rd^ visit, whereas the response in the VTG showed a significant reduction in symptoms in both regions at the 4^th^ visit	VAS for pain and pruritus	Pain: head/neck (*n* = 17), trunk (*n* = 19), upper limb (*n* = 6), lower limb (*n* = 1)
					Pruritus: head/neck (*n* = 19), trunk (*n* = 20), upper limb (*n* = 4), lower limb (*n* = 8)
					Total no. keloid patients: 78
Bijlard et al., 2018	History of recurrence	A: IL cryotherapy + excision + IL TCA; B: IL cryotherapy + excision + brachytherapy	History of recurrence: IL cryotherapy resulted in 40% reduction in scar volume in treatment naïve keloids, compared with 1% reduction in recalcitrant keloids	Keloid volume	Primary keloid: excision with TCA (*n* = 5), IL cryotherapy (*n* = 5)
					Recalcitrant keloid: excision with brachytherapy (*n* = 7), IL cryotherapy (*n* = 9)
					Total no. keloid patients: 26
Davison et al., 2006	Ethnicity, location	(Postoperative) A: IL interferon alpha-2b; B: IL TCA	Ethnicity and location: NS.	No. recurrences	Ethnicity: African American (*n* = 21), Caucasian (*n* = 13), Hispanic (*n* = 4), Asian (*n* = 1)
					Location: ear (*n* = 10), face/scalp (*n* = 8), chest (*n* = 7), extremity (*n* = 6), abdomen (*n* = 4), neck (*n* = 4)
					Total no. keloid patients: 34
Hewedy et al., 2022	Duration	A: IL TCA + PRP; B: IL TCA	Duration: NS.	VSS score	Mean duration (months): 15.8 (*n* = 20); 16.5. (*n* = 20)
					Total no. keloid patients: 40
Ismail et al., 2021	Duration, size	A: IL BTX-A; B: IL 5-FU	Size: NS in groups receiving BTX-A. However, small and medium lesions in the group receiving IL 5-FU showed a significantly better response than larger lesions	Keloid height	Duration: ≤2 years (*n* = 43), >2 years (*n* = 26)
			Duration: NS.		Size: small <1 cm^3^ (*n* = 43), medium 1–5 cm^3^ (*n* = 20), large >5 cm^3^ (*n* = 6)
					Total no. keloid patients: 50
Khan et al., 2019	Baseline POSAS, duration skin type	A: IL bleomycin; B: IL TCA	Fitzpatrick skin type, keloid duration, and baseline POSAS score: NS.	POSAS score	Mean duration (months): 4 (*n* = 164)
					Skin type: type 2 (*n* = 31), type 3 (*n* = 63), type 4 (*n* = 54), type 5 (*n* = 16)
					Mean POSAS baseline: 90 (*n* = 82), 91 (*n* = 82)
					Total no. keloid patients: 164
Manzoor et al., 2022	Duration	A: IL 5-FU; B: IL TCA alone, C: IL TCA + 5-FU	Duration: NS.	VSS score	Mean duration (months): 5.03 (*n* = 30), 6.30 (*n* = 30), 5.27 (*n* = 30)
					Total no. keloid patients: 90
Mourad et al., 2016	Duration, etiology, location	A: IL cryotherapy; B: cryospray	Duration: negative correlation between keloid duration and treatment efficacy	Swada and Sone scoring	Mean duration (months): NR
			Etiology and location: NS.		Etiology: acne (*n* = 16), burn (*n* = 18), surgery (*n* = 7), trauma (*n* = 8), vaccine (*n* = 2)
					Location: ear (*n* = 10), face/scalp (*n* = 8), chest (*n* = 7), extremities (*n* = 6), abdomen (*n* = 4), and neck (*n* = 4)
					Total no. keloid patients: 50
Neinaa et al., 2021	Baseline VSS, duration, size	A: IL BTX-A; B: IL PRP; C: IL TCA	Duration and size: NS.	VSS score	Mean duration (months): 5.2 (*n* = 20), 8.4 (*n* = 20), 7.4 (*n* = 20)
			Baseline VSS: higher baseline VSS scores were significantly correlated to better treatment outcomes in all studied groups (BTX-A and PRP were the most efficacious treatments)		Mean baseline VSS: 9.4 (*n* = 20), 9.7 (*n* = 20), 8.8 (*n* = 20)
					Mean size (cm^2^): 7.6 (*n* = 20), 8.4 *n* = 20), 7.4 (*n* = 20)
					Total no. keloid patients: 60
Rani et al., 2022	Duration, location	A: IL TCA, B: IL 5-FU, C: cryotherapy + IL TCA, D: surgical excision + topical 5% imiquimod	Duration: negative correlation between keloid duration and treatment efficacy. Lesions of <2 years showed better efficacy than lesions of >2 years (*p* < .05)	Unclear	Duration: <1 yr (*n* = 42), 1–2 year (*n* = 16), >2 year (*n* = 22)
			Location: “excellent response” on earlobes, face, and back. Poor efficacy on chest and shoulder (*p* < .05)		Location: chest (*n* = 38), earlobes (*n* = 16), shoulders (*n* = 11), face (*n* = 1)
					Total no. keloid patients: 80
Saha et al., 2012	Duration, location, no. of lesions	A: IL 5-FU; B: IL TCA	Duration, location and number of lesions: NS.	Keloid volume	Duration: ≤2 years (*n* = 22), > 2 years (*n* = 22)
					No of lesions (range): 1–6 (*n* = NR)
					Locations: arms (*n* = NR), back (*n* = NR), chest (*n* = NR)
					Total no. keloid patients: 44
Serag-Eldin et al, 2021	Duration, thickness	A: IL TCA, B: IL pentoxifylline, C: IL TCA + IL pentoxifylline	Duration and thickness: NS.	VSS score	Mean duration (months): 7.0 (*n* = 10), 7.2 (*n* = 10), 12.4 (*n* = 10)
					Mean thickness (cm): 2.9(*n* = 10), 4.4(*n* = 10), 3.0 (*n* = 10)
					Total no. keloid patients: 30
Tawfic et al., 2020	Duration, location	A: fractional CO2; B: Nd:YAG laser; C: CO2 + Nd:YAG lasers	Duration: significant negative correlation between keloid duration and treatment efficacy with Nd:YAG laser (NS for fractional CO2 laser or a combination of fractional CO2 laser followed by Nd:YAG laser)	VSS score	Mean duration (years): 8.84 (*n* = 30)
			Location: NS.		Location: upper extremities (*n* = 13), lower extremities (*n* = 5), trunk, 9 lower extremities + trunk (*n* = 3)
					Total no. keloid patients: 30

BTX-A, Botulinum toxin type A; 5-FU, 5-fluorouracil; duration, duration prior to treatment; IL, intralesional; No, number; NR, not reported; NS, no significant correlation with treatment efficacy; POSAS, patient and observer scar scale; PRP, platelet-rich plasma; TCA, triamcinolone acetonide; VAS, visual analogue scale; VRS, verbal rating scale; VSS, Vancouver Scar Scale; VTG, verapamil treatment group.

### Keloid Duration Prior to Treatment

Twelve studies involving 811 patients investigated keloid duration prior to treatment.^3–14^ Three studies reported higher efficacy in younger keloids compared to longer existing keloids.^7,9,12^ Rani and colleagues^12^ reported higher efficacy in younger keloids (<2 years), however, without performing subanalysis per treatment group (intralesional triamcinolone acetonide [il TCA]; il 5-FU; il TCA + cryotherapy; surgical excision + topical imiquimod). The 2 other studies did not report a specific cut-off point for duration but reported significantly higher efficacy in younger keloids treated with respectively Nd:YAG laser or cryotherapy.^7,9^ The remaining studies found no significant correlation.^3,4,6,8,10,11,13,14^

### Keloid Location

Seven studies involving 440 patients investigated keloid location.^7,9,12,13,15–17^ One study found significantly higher “cure rates” for keloids treated with il TCA that were located on the cheek, forehead, submandibular area, and lip compared to the pinna, while no subanalysis was reported for keloids treated with excision and radiotherapy.^15^ Belie and colleagues^16^ reported higher efficacy for keloids located on the trunk compared to the extremities after treatment with il TCA or il verapamil. Rani and colleagues^12^ reported higher efficacy for keloids located on the earlobes, face, and back, compared to the chest and shoulders, without a subanalysis per treatment group (il TCA; il 5-FU; il TCA + cryotherapy; surgical excision + topical imiquimod). The other studies found no significant correlation.^9,14,15,17^

### Keloid Size

Three studies involving 195 patients investigated keloid size.^3,5,8^ Two studies reported higher efficacy in smaller keloids (<1 cm^2^ and <5 cm^3^) compared to larger keloids after respectively contact cryosurgery or il 5-fluorouracil.^3,5^ The other study found no significant difference between smaller and larger keloids.^8^

### History of Recurrence

One study involving 26 patients investigated treatment history.^18^ After intralesional cryotherapy, a volume reduction of 40% versus 1% was observed in respectively naïve versus recurrent (previous corticosteroid injections or excision) keloids.

### Baseline Vancouver Scar Scale Core

One study involving 60 patients investigated baseline Vancouver Scar Scale (VSS).^8^ Higher efficacy was reported in keloids with higher baseline VSS scores compared to keloids with lower baseline VSS scores. However, no specific cut-off points for VSS and subanalysis per treatment group (il botulinum toxin type-A, il platelet rich plasma, and il TCA) were mentioned.

### Other Keloid Properties

Fitzpatrick skin type (*n* = 2; 324 patients),^4,6^ baseline Patient & Observer Scar Assessment Scale (POSAS) score (*n* = 1; 164 patients),^6^ ethnicity (*n* = 1; 39 patients),^17^ etiology (*n* = 1; 50 patients),^7^ keloid thickness (*n* = 1; 90 patients),^11,14^ and number of lesions (*n* = 1; 44 patients)^13^ did not show a significant correlation with treatment efficacy.

### Risk of Bias Assessment

**Figure 2.**
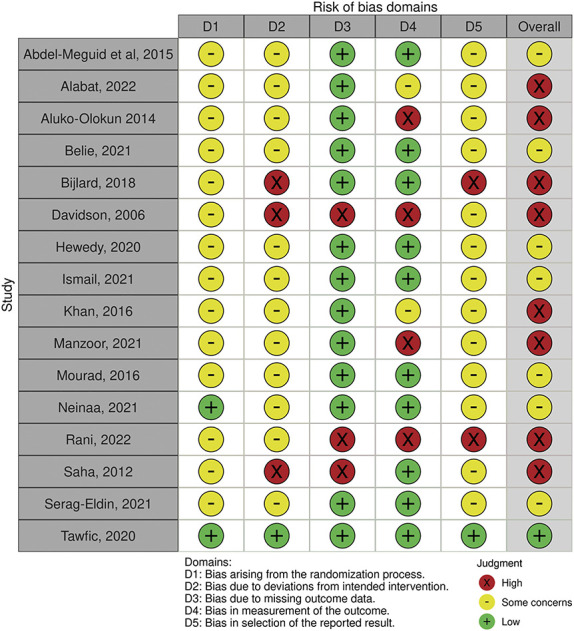
Risk of bias of the included RCTs. Half of the articles (50%) were judged as having “high risk of bias.” One article (6%) was assessed as having “low risk of bias,” and the remaining articles (44%) were judged as having “some concerns.”

The overall risk of bias was rated as “high” in 8 studies, “some concerns” in 7 studies, and “low” in 1 study (Figure 2). Methodologic quality was particularly poor due to bias arising from the randomization process, deviations from the intended intervention, and selective reporting.

## Discussion

This systematic review aimed to identify clinically relevant keloid properties that may impact treatment efficacy. In total, only 16 RCTs performed a separate analysis for specific keloid properties. In these 16 studies, keloid duration prior to treatment and location were the most frequently analyzed properties followed by size, Fitzpatrick skin type, baseline POSAS score, baseline VSS score, keloid thickness, ethnicity, etiology, number of keloids, and history of recurrence.

The authors' findings suggest a lower treatment efficacy in keloids with a longer duration prior to treatment, location on the chest, extremities, pinna, or shoulder, a larger size, history of recurrence, and a lower baseline VSS score of keloids. However, the authors cannot exclude clinical relevance of the other keloid properties because the number, sample size, and quality of the studies are insufficient to draw firm conclusions. Hence, more research is needed with a focus on these keloid properties.

Some keloid properties were found to be relevant for specific treatments only, suggesting that the influence of keloid properties depends on the treatment used. In line with this finding, some experts proposed treatment algorithms addressing specific keloid properties.^19,20^ Although size and number of keloids were mentioned in these algorithms as important properties to take into account, other potentially clinically relevant properties such as duration prior to treatment and location of keloids were not mentioned. Moreover, these algorithms were not based on a systematic review.

Importantly, a diversity of outcome instruments and scales were used in the included studies. For instance, some studies used the Swada and Sone,^7^ POSAS,^4,6^ or VSS scale ^8,9^ to evaluate keloid treatment outcomes, while others used reduction in keloid size^3,5,18^ as the primary outcome. This variation in outcome measures makes it challenging to compare results between studies, decreasing the value of these studies, and contributing to waste in research. Therefore, it is imperative that a consensus-based Core Outcome Set will be implemented in future research and reporting in this field.

The strengths of this systematic review include the use of a comprehensive database search, inclusion of RCTs with no limitation of publication date, and a critical methodologic quality assessment using the ROB 2.0. Limitations of this review include the low number of eligible studies and the heterogeneity of outcome measures and scales, which precludes a meta-analysis. Moreover, the study populations were generally small, which makes it difficult to detect differences in efficacy between keloid properties.

In conclusion, only a minority of studies performed subanalyses for specific keloid properties and even fewer studies found clinically relevant keloid properties. The authors' results suggest that keloid duration prior to treatment, location, size, history of recurrence, and severity influence treatment efficacy. Nonetheless, more high quality head-to-head RCTs using validated outcome measures should report on the potentially relevant keloid properties. These further investigations are crucial to corroborate the authors' findings, establish a clinically relevant keloid classification, and ultimately develop an evidence-based treatment algorithm for clinical practice that takes these properties into account.
